# A Rapid and Simple Bioassay Method for Herbicide Detection

**DOI:** 10.4137/bmi.s594

**Published:** 2008-04-28

**Authors:** Xiu-Qing Li, Alan Ng, Russell King, Dion G. Durnford

**Affiliations:** 1 Agriculture and Agri-Food Canada, 850 Lincoln Road, P.O. Box 20280, Fredericton, NB, E3B 4Z7, Canada; 2 Department of Biology, University of New Brunswick, Fredericton, NB, E3B 6E1, Canada

**Keywords:** herbicidal activity detection, run-off, Bioassay, paper-disk

## Abstract

*Chlamydomonas reinhardtii*, a unicellular green alga, has been used in bioassay detection of a variety of toxic compounds such as pesticides and toxic metals, but mainly using liquid culture systems. In this study, an algal lawn—agar system for semi-quantitative bioassay of herbicidal activities has been developed. Sixteen different herbicides belonging to 11 different categories were applied to paper disks and placed on green alga lawns in Petri dishes. Presence of herbicide activities was indicated by clearing zones around the paper disks on the lawn 2–3 days after application. The different groups of herbicides induced clearing zones of variable size that depended on the amount, mode of action, and chemical properties of the herbicides applied to the paper disks. This simple, paper-disk-algal system may be used to detect the presence of herbicides in water samples and act as a quick and inexpensive semi-quantitative screening for assessing herbicide contamination.

## Introduction

Algal liquid cultures have commonly been used as a bioassay system for toxicological tests on water samples for the assessment of herbicide action (Eberle and Gerber, 1976; Wundram et al. 1996; Brack et al. 1998; Fairchild et al. 1998; Devez, 2004) and the presence of toxic metals (Overnell, 1975; Rojickova-Padrtova and Marsalek, 1999; Devez et al. 2005). An agar-based, paper disk approach, however, has the advantages of being a rapid, easy and cheap screening approach and is based on approaches developed for bacteria (Gavin, 1957). Such an approach has been effective for studying the growth inhibiting properties of Actinomycetes (Safferman and Morris, 1962) and antibiotics (Andreozzi et al. 2006) on cyanobacteria. In the present study, we developed an *algal-lawn and paper-disk* system using *C. reinhardtii* as an inexpensive and rapid procedure for testing the feasibility of assessing activities of all the current 11 groups of herbicides with the potential application of screening leachate toxicity.

## Materials and Methods

### Herbicides

Sixteen herbicides were purchased from commercial suppliers for this study: Acifluorfen, Chlorpropham, Diclofop-methyl (DFM), Glyphosate, Isoxaben, Pinnacle, and Trifluralin (Coledon Laboratories, QC, Canada); dichlorobenzonitrile (DCB) (Sigma, Mo); 2,4-dichlorophenoxyacteic acid (2,4-D), Metobromuron, 2-ethyl-4-chlorophenoxyacetic acid (MCPA), Metribuzin, Atrazine, Hexazinone (Riedelde Haen, Germany); Norflurazon (Sandoz Agro, IL); and Terbacil (Du Pont Sinbar, DE). All the stock solutions were prepared with 95% ethanol in water and stored at 4 °C in the dark and used within two months.

### Preparation of the *Chlamydomonas* lawn

*Chlamydomonas reinhardtii* (Strain CC125, *Chlamydomonas* Stock Centre, Duke University) was grown in Tris-Acetate-Phosphate (TAP) media (Harris, 1989) under continuous light (cool white fluorescent) at 300 μmol quanta m^−2^sec^−1^ and 25 °C to a density ≥5 × 10^6^ cells/ml. A total of 4 × 10^7^ cells were concentrated by centrifuging at 900 × g for 5 minutes and the cell pellet re-suspended in 0.5 ml fresh TAP media. The concentrated cells were quickly mixed with 3 ml of top agar (0.5% agar in TAP medium) maintained at exactly 38 °C and poured immediately onto a 1.2%-agar TAP medium plate to form a solidified lawn of cells. 38 °C is near the maximum temperature tolerance for this organism so careful temperature control is important.

The disks (Millipore filter-type HA, 0.45 mM pore size) were cut to a 6 mm diameter using a standard paper hole punch to which herbicides were applied directly at three different concentrations (Table 1). In each case, the ethanol was allowed to evaporate thoroughly before addition of the disks to the lawn. Control disks had equal amounts of the appropriate solvent applied but lacking inhibitors. After the top agar had solidified, the dried disks containing the herbicides were placed on top of the *Chlamydomonas* lawn and the plates were inverted and returned to the growth chamber. Observations were taken three days after the application of the disks to the algal lawn. The experiments were repeated 4 times for the herbicides (trifluralin, 2,4-D, and MCPA) that produced only small clear zones around the disks, and twice for those that produced large clear zones.

The toxins/herbicides were added to the paper disks in different quantities as they all have unique optimal concentrations for inhibition in plants. As a guideline, the smallest concentration added to the plate (Disk 1) was based on the minimal amount of herbicide equivalent to regulatory Health Advisory Level-Maximum Contaminant Level (HAL-MCL, http://www.epa.gov) or run-off levels. The calculations of concentration were based on a 5 ml volume as the total TAP agar applied to the Petri plate was 30 ml and there were a total of 6 disks (3 treatments and 3 controls) added per plate. In most cases, the amount of each chemical tested increased five and 25 fold from the basal amount loaded onto Disk 1 for Disks 2 and 3, respectively (Table 1).

## Results and Discussion

**Figure 1:**
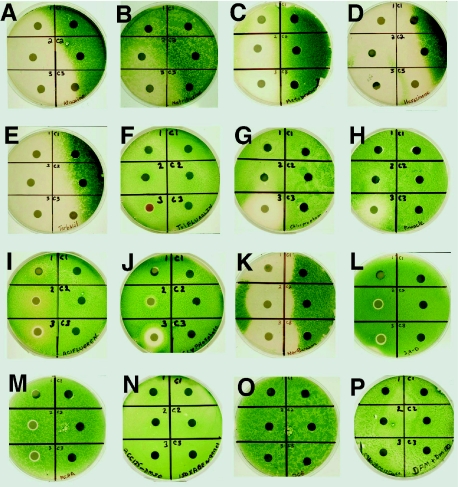
Clearing zones produced by the herbicides around the paper disks indicating the inhibition of the growth of *C. reinharditii* cells. The three paper disks on the left in each petri-dish are the treatments (disk 1–3); The three paper disks on the right are controls without herbicides. The herbicide treatments are the followings: **A**, Atrazine; **B**, Metribuzin; **C**, Metobromuron; **D**, Hexazinone; **E**, Terbacil; **F**, Trifluralin; **G**, Chlorpropham; **H**, Pinnacle; **I**, Acifluorfen; **J**, Glyphosate; **K**, Norflurazon; **L**, 2,4-D; **M**, MCPA; **N**, Isoxaben; **O**, DCB; **P**, DFM.

The disk bioassay is a convenient, diffusion-based approach to examining the biological effects of any number of compounds and has been in use for decades (Gavin, 1957). Our modification of this technique optimizes it for use in a readily available, model algal system for the purposes of screening for herbicidal activities. *Chlamydomonas* was sensitive to a broad range of commonly used herbicides and the sensitivity of the alga towards each is indicated by the zone of clearing around the disks (Fig. 1).

Photosynthetic inhibitors that block at Photosystem II (Atrazine, Metobromuron, Hexazinone, and Terbacil) were very effective in inhibiting algal growth and were easily detected in this assay at typical maximal regulatory concentrations (Fig. 1, A, C–E). Metribuzine is another PSII inhibitor but it was less effective in inhibiting algal growth and clear herbicidal activity was detected at levels ≤ 5 times greater than typical regulatory levels (Fig. 1B). The effects of other classes of herbicides were also easily detected in this assay, including inhibitors of microtubule assembly (Chlorpropham, Fig. 1G) acetolacetate synthase (Pinnacle, Fig. 1H), protoporphyrinogen oxidase (Acifluorfen, Fig. 1 I), EPSP synthase (Glyphosate, Fig. 1J), and inhibitors of carotenoid biosynthesis (Norflurazon, Fig. 1K) (Brack et al. 1998). *C. reinhardtii* was also sensitive to auxin analogues (2,4-D and MCPA, Fig. 1L, M), but at higher concentrations. Among all the 16 herbicides tested, isoxaben, DCB and DFM (Fig. 1N, O, P , respectively) were unable to inhibit growth. Isoxaben and DCB belong to a class of herbicides that inhibit plant cell wall (cellulose) synthesis (Nakagawa and Sakurai, 1998; Delmer, 1999). Considering that *C. reinhardtii* does not have a cellulose-based cell wall (Harris, 1989), these results are not surprising. The reason for the resistance of *C. reinhardtii* to Diclofop-methyl at the tested concentrations is not clear.

The clearing zones around each disk had different characteristics for each group of herbicides tested, which is ultimately dependent upon the rate of diffusion and the mode of action of the herbicide. *C. reinhardtii* cells were most sensitive to inhibitors of photosynthesis (Fig. 1 A–E) or those that blocked chlorophyll or carotenoid biosynthesis (Fig. 1 I, M). These herbicides produced large clear zones where algal growth was inhibited and cells rapidly died. The auxin analogues (Fig. 1L and M) produced narrow but very clear circular zones around the disks containing the herbicides, but only at concentrations 5 times greater than typical allowable contamination levels. This indicates that these auxin analogues have low diffusion rates and are inhibitory above a high threshold concentration. The significance of the observed effect of the auxin-type herbicides in a unicellular alga is unknown but intriguing. Other types of herbicides that inhibit cell growth showed intermediate effects that included cleared zones with a halo of reduced growth.

The simple unicellular algal system was of use in comparing the effectiveness of herbicides with known mode of actions, with the size of the clearing zone around the disk being a semi-quantitative estimation of the amount of herbicide loaded to the disk. Such a simple and inexpensive assay could be useful for the detection of herbicidal activities in runoff and groundwater samples. In this case, residues from the water sample would have to be concentrated to a predetermined volume that represents the maximal permissible runoff levels and applied to the disk to assess the presence of toxic materials. By reducing the thickness of the agar base, the amount of sample that would have to be concentrated could be reduced to 1–2 ml, resuspended in a small volume of 95% ethanol, loaded onto the disc and placed upon the algal lawn. In our tests of ground water, the salts and minerals left behind after evaporation of 1 ml where minimally soluble in 95% ethanol and had no effect on algal growth using this assay. The fact that different herbicides of the same class gave similar results in the tests indicated that the disk diffusion-algal lawn method is reliable for detecting the presence of herbicidal activity. Variations of this approach have previously been used on algal systems to examine the biological activities of a variety of compounds (Safferman and Morris, 1962; Andreozzi et al. 2006). This assay could also be applied to the detection of other environmental contaminates including heavy metals (Wundram et al. 1996) and a variety of toxins in landfill leachates (Brack et al. 1998) although the appropriate controls will be needed to account for any variations in water chemistry including salt concentration and pH range of different water sources. A positive result using concentrated water samples would indicate the presence of potential toxins, but the method may find utility as a rapid screen to identify samples requiring more detailed examination.

## Figures and Tables

**Table 1:** Results obtained for each herbicide in the *Chlamydomonas reinhardtii* growth inhibition tests.

Herbicide	Site of Inhibition/Mode of Action	Amount (mg)			Results
		Disk 1	Disk 2	Disk 3	
2,4-D^O^	Synthetic auxin	0.5	2.5	5	Sensitive
Acifluorfen^E^	Protoporphyrinogen oxidase	0.25	1.25	6.25	Sensitive
AtrazineC1	PSII	0.0005	0.0025	0.0125	Sensitive
ChlorprophamK2	Microtubule polymerization	0.004	0.02	0.1	Sensitive
DCB^L^	Cell wall synthesis (site A)	0.085	0.425	2.125	No effect
Diclofop-methyl^A^	Acetyl CoA carboxylase	0.625	3.125	15.625	No effect
Glyphosate^G^	EPSP synthase	0.625	3.125	15.625	Sensitive
HexazinoneC1	PSII	0.003	0.015	0.075	Sensitive
Isoxaben^L^	Cell wall synthesis (site B)	0.085	0.425	2.125	No effect
MCPA^O^	Synthetic auxin	0.5	2.5	5	Sensitive
MetobromuronC2	PSII	0.5	2.5	5	Sensitive
MetribuzinC1	PSII	0.05	0.25	1.25	Sensitive
NorflurazonF1	Phytoene desaturase	0.004	0.02	0.1	Sensitive
Pinnacle^B^	Acetolactate synthase	0.005	0.025	0.125	Sensitive
TerbacilC1	PSII	0.0005	0.0025	0.0125	Sensitive
TrifluralinK1	Microtubule assembly	0.188	0.938	4.688	Sensitive

A, B, C1, C2, E, F, G, K1, K2, L, O Herbicides classified according to the herbicide groups classified by the international Herbicide Resistance Action Committee (Schmidt, 1997). PSII: Photosystem II complex. EPSP synthase: 5-enolpyruvylshikimate-3-phosphate synthase. Note that DCB and Isoxaben inhibit cellulose-based cell walls, which *C. reinhardtii* does not have.
